# Liposomes as Antibiotic Delivery Systems: A Promising Nanotechnological Strategy against Antimicrobial Resistance

**DOI:** 10.3390/molecules26072047

**Published:** 2021-04-02

**Authors:** Magda Ferreira, Maria Ogren, Joana N. R. Dias, Marta Silva, Solange Gil, Luís Tavares, Frederico Aires-da-Silva, Maria Manuela Gaspar, Sandra Isabel Aguiar

**Affiliations:** 1 Centro de Investigação Interdisciplinar em Sanidade Animal, Faculdade de Medicina Veterinária, Universidade de Lisboa, Avenida da Universidade Técnica, 1300-477 Lisbon, Portugal; magdaferreira@fmv.ulisboa.pt (M.F.); mlso93@hotmail.com (M.O.); joananrdias@fmv.ulisboa.pt (J.N.R.D.); mlsilva@fmv.ulisboa.pt (M.S.); solange@fmv.ulisboa.pt (S.G.); ltavares@fmv.ulisboa.pt (L.T.); fasilva@fmv.ulisboa.pt (F.A.-d.-S.); 2 Research Institute for Medicines (iMed.ULisboa), Faculty of Pharmacy, Universidade de Lisboa, Av. Prof. Gama Pinto, 1649-003 Lisbon, Portugal

**Keywords:** liposome, antibiotic, bacterial infection, antimicrobial resistance

## Abstract

Antimicrobial drugs are key tools to prevent and treat bacterial infections. Despite the early success of antibiotics, the current treatment of bacterial infections faces serious challenges due to the emergence and spread of resistant bacteria. Moreover, the decline of research and private investment in new antibiotics further aggravates this antibiotic crisis era. Overcoming the complexity of antimicrobial resistance must go beyond the search of new classes of antibiotics and include the development of alternative solutions. The evolution of nanomedicine has allowed the design of new drug delivery systems with improved therapeutic index for the incorporated compounds. One of the most promising strategies is their association to lipid-based delivery (nano)systems. A drug’s encapsulation in liposomes has been demonstrated to increase its accumulation at the infection site, minimizing drug toxicity and protecting the antibiotic from peripheral degradation. In addition, liposomes may be designed to fuse with bacterial cells, holding the potential to overcome antimicrobial resistance and biofilm formation and constituting a promising solution for the treatment of potential fatal multidrug-resistant bacterial infections, such as methicillin resistant *Staphylococcus aureus*. In this review, we aim to address the applicability of antibiotic encapsulated liposomes as an effective therapeutic strategy for bacterial infections.

## 1. Introduction

Antimicrobials are indispensable pharmaceuticals to treat bacterial infections. However, for decades, antibiotics have been overused in clinic, agriculture and animal production setting, generating a strong selection pressure over bacterial species [1,2]. The ultimate consequence has been the emergence and dissemination of antimicrobial resistant strains among humans, animals and the environment, culminating in the rise of the global health problem of antibiotic resistance, one of the top major threats to global public health of the 21st century [3].

Measures to control and overcome antibiotic resistance are urgently needed to avoid a clinical collapse which might be over the edge. Limiting the use of antibiotics while improving hygiene and antibiotic disposal measures have been the main action plans proposed by the World Health Organization (WHO) and governmental health agencies [4,5]. In parallel, significant funding is being allocated to the development of new and effective strategies against multidrug resistant (MDR) bacteria, with biological, adjuvant therapies, phages and small molecules with anti-quorum, anti-bacterial virulence or anti-toxin effects expanding the panel of potential therapeutic strategies [6]. However, the research and development of new antibiotics, biologicals or adjuvant therapies is a laborious process which cannot keep pace with the increasing rates of drug resistance and the urging need of fast-track approved approaches [7].

Taking this into account, an appealing alternative to the search of new therapies is the combination of currently approved antibiotics with the use of nanotechnology, also known as “nanobiotics”. Recent advances in this field have enabled the development of drug delivery systems with improved antimicrobial features and pharmacokinetic profiles [8,9,10]. These biomedical nanotechnology systems are remarkably improving the therapeutic effects of conventional drugs and may hold the promise of changing the efficacy of currently available antibiotics [11]. Among the wide array of nanoplatforms, one of the most promising delivery approaches for antibiotics under investigation are liposomes [12,13]. These lipid-based nano-systems were introduced as drug carriers in the 1970s, and, since then, major breakthroughs in liposome technology have driven the interest of their use as efficient delivery systems for antibacterial drugs [13,14,15].

The focus of this review aims to highlight the advantages of liposomes as carriers of antimicrobial agents and their potential to not only eradicate the infection but also overcome antibiotic resistance [16]. Hence, in an era of a massive increase of infections due to multidrug resistant bacteria, the use of antibiotic incorporated liposomes is a potential alternative to circumvent the limitations of conventional antimicrobial therapies [17,18].

## 2. Nanotechnological Approaches for Treatment of Bacterial Infections

Multiple nano-sized vesicles, such as metallic nanoparticles, liposomes, dendrimers, polymeric nanoparticles and carbon nanotubes, have been designed with improved therapeutic properties of the incorporated compounds, such as controlled release, decreased systemic toxicity, drug-targeting and higher efficiency [19].

Within these nanotechnological-based approaches available, lipid-based nano-systems such as liposomes have demonstrated particularly appealing features in terms of physicochemical properties and safety issues. Liposomes are vesicular concentric bilayer structures composed of relatively biocompatible and biodegradable materials. They offer several advantages over other delivery systems due to their unique characteristics to incorporate hydrophilic and hydrophobic drugs, biocompatibility, biodegradability, low toxicity and lack of immune system activation [14,20,21]. In addition, liposomes can be easily coupled with targeting platforms, such as antibodies, proteins or enzymes, thus allowing a specific delivery of bioactive compounds directly into infection sites [22,23,24,25]. More importantly, as detailed in Table 1, several liposomal-based formulations have been approved by the FDA for clinical use or are in clinical trials in the infectious disease setting, demonstrating their therapeutic potential and the possibility of fast-track approval of subsequent formulations.

## 3. Structure and Properties of Liposomes

**Figure 1:**
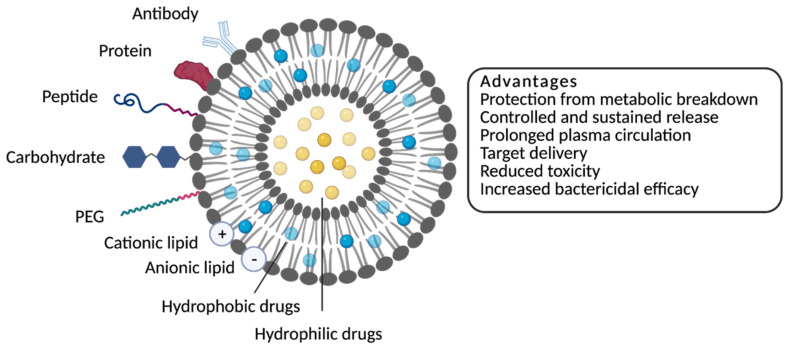
Schematic representation of different types of liposomes and their major advantages.

Liposomes are small, spherical vesicles composed of one or more phospholipid bilayers surrounding aqueous compartments or units (Figure 1) [20,26]. They are distinguished from other nanoparticles due to their ability to encapsulate hydrophilic drugs within the aqueous compartment and/or hydrophobic drugs inside the lipid bilayer, greatly increasing the diversity of drugs that can be incorporated [27,28]. Liposomal formulations are generally characterized by size (small unilamellar vesicles (SUVs) < 100 nm, large unilamellar vesicles (LUV) > 100 nm), number of lamellae (unilamellar or multilamellar vesicles), lipid composition, charge of the bilayer (anionic, cationic or neutral) and surface functionalization with polymers or ligands. These properties are known to influence their stability and biological performance both in vitro and in vivo [25,29]. Charge and composition are particularly important features since they arbitrate the fluidity and stability of the liposomal membrane and impact the liposome–bacteria interaction [20,21,26,29]. An essential characteristic that upraises liposomes to potential disruptive antibacterial therapeutics is, as more deeply discussed below, their flexibility for surface modification. The surface functionalization, as detailed in Figure 1, with ligands, such as polymers (e.g., PEGylated liposomes) and molecules (e.g., antibodies, proteins/peptides and carbohydrates), is used for specific targeting (ligand-targeted liposomes) [12,29], playing an important role in efficient delivery and therapeutic efficacy.

## 4. Advantages of Liposomes as Antibiotic Carriers

**Figure 2:**
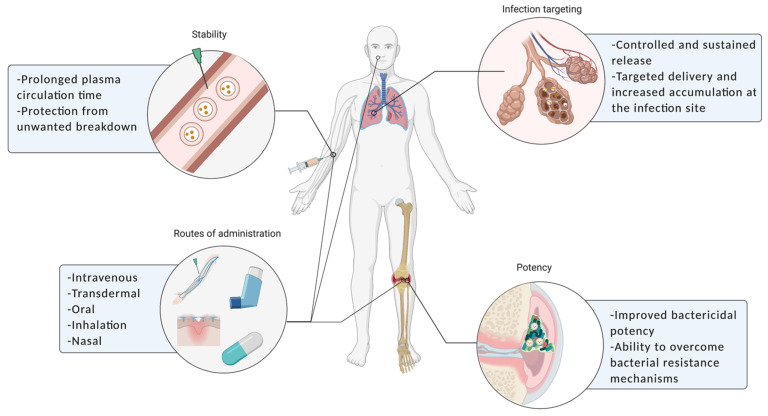
Schematic representation of the main advantages of liposomes as antibiotic carriers.

Recent improvements in liposomal formulations have enabled the development of potential antibiotic delivery platforms that could revise critical issues in the treatment of infectious diseases. As described above, liposomes have several advantages as antibiotic delivery nanosystems, overcoming the problems related either with efficacy of the incorporated drug or selection of resistant strains. Several studies have shown that liposomal encapsulation promotes the stability and safety of antibiotics, giving rise to more appropriate pharmacokinetic and pharmacodynamic profiles by prolonging the circulation time in bloodstream, enabling a specific targeting to the infection sites using different routes of administration (Figure 2) [22,30].

### 4.1. Stability

#### 4.1.1. Controlled and Sustained Release of Antibiotics

Bioavailability and antibiotic concentration at the infection site is highly dependent on the administration route (intravenous, oral and pulmonary), class of antibiotics, dosing and treatment duration, drug interaction, co-morbidities and overall patient condition (particularly in critically ill patients) [31,32,33]. This variability impacts not only the infection clearance rate but also may contribute to the development of antimicrobial resistance since only a fraction of the antibiotic actually reaches its target. For this reason, when free drugs are administered, high and repeated dosing is indispensable to maintain antibiotic concentrations above the bacteria minimum inhibitory concentration (MIC) [33].

Nevertheless, several studies have demonstrated that, for drugs that present concentration dependent activity, such as fluoroquinolones and aminoglycosides, higher antibiotic dosages are more efficient in clearing and preventing the selection of resistant strains than fractionating regimens [34,35]. However, the use of high concentrations is limited by dose-dependent toxic effects of the antibiotics, which makes the management of the therapeutic dosing of an infection, caused by an antimicrobial resistant, a clinical challenge [32].

A major advantage of incorporating antibiotics in liposomes is the possibility to regulate the release of the entrapped antibiotic. Depending on their composition and presence of specific stimulatory factors, such as pH or heat, liposomes can be engineered to disintegrate and subsequently release incorporated drugs in a controlled manner [21,29]. This may occur only at the infection site, without premature release during circulation or be sustained over a period of time enabling the reduction of dosing frequency and consequent systemic toxicity [13,36,37,38]. The sustained release of antibiotics may be, by itself, highly beneficial against antimicrobial resistant strains since it enables the maintenance of a higher than MIC concentration without secondary toxicity.

#### 4.1.2. Prolonged Plasma Circulation Time

The dose regimen of antibiotics will depend on the pharmacodynamic properties and mode of action of the antibiotic itself. Some antibiotics, such as β-lactams and vancomycin, present slow bactericidal activity. In this case, the antimicrobial action has a high time-dependence and minor improvements in bactericidal action are attained using higher concentrations. As such, frequent dosing is necessary in order to maintain the antibiotic serum concentration within therapeutic levels [35]. Furthermore, some classes of antibiotics (such as β-lactams) have increased serum protein binding features, influencing the free antibiotic concentration in plasma and impairing the antibacterial activity [39].

Antibiotic encapsulation in liposomes can provide a therapeutic improvement by increasing the circulation time, allowing a higher antimicrobial efficacy without the need of repeated administrations or high dosing concentrations. Systemic circulation time of liposomes can be increased by manipulating the physicochemical properties of the liposome, such as size and surface charge, with neutral and/or small-size liposomes presenting longer circulation half-life [21,40]. Some liposomes, containing natural or synthetic phospholipids, undergo high systemic clearance by the mononuclear phagocytic system (MPS) and accumulate preferentially in liver and spleen [20,41,42,43]. To avoid MPS uptake, they can be coated with biocompatible hydrophilic polymers such as polyethylene glycol (PEGylated liposomes). This strategy enhances liposome stability, reduces the interaction with plasma proteins, decreases recognition by macrophages and increases circulation time in bloodstream [44,45,46]. Furthermore, the encapsulation in liposomes allows protection from unwanted enzymatic degradation and from chemical or immunological deactivation [23]. Indeed, improvement in circulation time has been observed in PEGylated liposomes encapsulating ciprofloxacin and vancomycin, leading to significantly higher serum concentrations when compared to the administration of the respective free antibiotic [47,48].

### 4.2. Infection Targeting

Undoubtedly, the perfect antimicrobial treatment should preferentially allow clearance of the infectious agent without disturbing the essential microbiota or adjacent cells. Considering the available antimicrobial resources, this goal is far from being achievable for bacterial infections. Nevertheless, a more realistic approach is the development of antibiotic-loaded targeting vehicles. Directing to a specific site, such as a selective tissue, organ or eventually a strictly defined pathogenic bacteria, is a key functional aspect of liposomes and one of the most appealing features for the delivery of antimicrobial agents [13,18,49].

Targeting infection sites can be achieved either by direct administration of the antimicrobial agent at affected areas or by developing drug delivery strategies with organ or bacterial marking features. In particular types of infection, such as respiratory infections, the pulmonary route is more appealing in terms of ease of administration and patient compliance. In fact, aerosol antimicrobial therapy is gaining increased clinical interest mainly due to the superior antimicrobial activity, particularly for antibiotics with poor lung penetration such as β-lactams, colistin, aminoglycosides and glycopeptides. Moreover, the immunomodulatory effect associated with a decreased systemic toxicity has led to the approval of several aerosol antibiotics in the last years, as reviewed by Zarogoulidis and collaborators [50].

Despite the improvements in inhalation delivery technologies, the majority of the formulations are still hampered by the short half-life and rapid clearance of the antibiotic from the lung, which could result in sub-inhibitory antibiotic concentrations and decreased antimicrobial efficacy [51,52]. To surpass this issue, liposomes are being developed to guarantee a sustained release of the antibiotics in aerosol formulations, allowing maintenance of antibiotic concentrations above MIC without the need for frequent dosing [52]. Antibiotic liposomal aerosol formulations have been developed for a variety of pathogens including *Pseudomonas aeruginosa* and *Mycobacterium* spp., with amikacin, ciprofloxacin and tobramycin being some of the antibiotics considered for this application, as reviewed by Basseti and collaborators [52]. Overall, significant improvements in bacterial clearance have been observed, for example, for inhaled ciprofloxacin liposomal formulations for both *P. aeruginosa* and non-tuberculosis mycobacteria [51,53,54]. In addition, to improve the efficacy of lung infection treatments due to MDR bacteria, the possibility of combining two antibiotics within the same liposomal formulation is being considered. The potential of a dry powder inhaler liposomal formulation combining colistin and ciprofloxacin has been developed and tested in vitro against clinical isolates of *P. aeruginosa* by Wang and collaborators. In this work, the authors observed a synergistic effect when using the two antibiotics [55] being particularly appealing for MDR infections.

Another example of direct administration is the topical delivery of liposomal antibiotics for ophthalmic and dermal applications. Antimicrobial preparations are preferentially applied locally to prevent and treat burn infections and non-healing chronic wounds such as diabetic, vascular and pressure ulcers [56]. Despite the accessibility to the skin and the eye, not all drugs incorporated in creams, ointments or suspensions are delivered in appropriate concentrations due to drug instability issues or body’s defense mechanisms. Moreover, the increasing prevalence of antibiotic resistant bacteria in skin infections, such as MRSA, which can disseminate to deeper soft tissues and lead to infections such as cellulitis, abscesses or even necrotizing fasciitis [57], is urging the need of more successful therapies. In line with this, a significant number of research studies have developed liposomal formulations for skin infections aiming to promote an effective clearance of the pathogen while contributing to skin regeneration and wound healing [58]. Price and collaborators developed silver sulfadiazine encapsulated in liposomes for *P. aeruginosa* soft tissue infections and observed that one single dose significantly decreased bacteria CFUs compared to multiple applications of the free drug [59].

Indeed, liposomal formulations for topical administration are already in the market. Liposomal polyvinyl-pyrrolidone (PVP)-iodine hydrogel is a commercial lipid-based formulation, used for wound healing. PVP-iodine is an antiseptic agent that, in addition to anti-inflammatory effect, has clinical activity against a wide range of antibiotic resistant bacteria. Taking this into account, Augustin et al. performed a pilot study aiming to evaluate the efficacy and tolerability of liposomal PVP-iodine of localized inflammatory dermatoses associated with bacterial colonization. This study revealed the absence of allergic reactions in patients with infective dermatosis treated with this liposomal formulation, in contrast to the reported cases from patients using PVP-iodine in the free form. Moreover, this formulation demonstrated a high therapeutic potential for several inflammatory skin disorders associated to bacterial infections. Liposomal PVP-iodine enhanced the disease-related symptoms and global clinical severity scores of acne vulgaris, atopic dermatitis, rosacea and impetigo contagious [60].

When the direct administration at the infected site is not possible, liposomal formulations can be designed to target the infection in non-specific or specific approaches. The non-specific interaction between liposomes and bacteria has been described after intravenous administration of liposomes. Depending on the physicochemical properties of the liposomes, in particular their surface charge, preferential accumulation at infected sites and direct interaction with bacteria have been reported [13,22,61]. Pathogenic bacteria possess, under physiological conditions, a negatively charged cell wall. Hence, positively charged liposomal vesicles are able to target bacteria by electrostatic interactions [18,62]. Stimuli responsive liposomes are other example of non-specific targeting. Liposomes can be designed to liberate its encapsulated compound in a pH or temperature dependent form. pH-sensitive liposomes are able to change their conformation and chemical properties in response to acidic pH. An example is the combination of dioleoyl phosphatidyl ethanol amine (DOPE) and cholesteryl hemisuccinate (CHEMS) in the lipid composition of liposomes, which promotes the destabilization of their lipid bilayer, releasing the incorporated molecule, when reaching a low pH microenvironment. Liposomes with pH-responsive features allow self-targeting and accumulation at infectious biofilms, characterized by having acidic pH [63]. Temperature-sensitive liposomes are able to release the incorporated drug in response to local heating [64]. Incorporated drugs are released from liposomes at temperatures above the melting transition temperature of the lipid bilayer [65].

To achieve a specific interaction, liposomes are coupled with targeting ligands at their surface, such as proteins, aptamers, antibodies or antibody fragments, that are recognized by particular surface receptors located at the target cells allowing a localized delivery of the liposomal formulation [66,67,68]. This may allow a reduction of the total dose required for treatment, and consequently decrease drug accumulation at healthy tissues and the risk of dose-dependent toxicity. This strategy is particularly interesting for antibiotics such as vancomycin for which nephrotoxicity limits their clinical usefulness [69]. Furthermore, this approach can also be used to develop liposomes for targeting difficult to treat intracellular bacterial infections caused by *Mycobacterium tuberculosis* or *Listeria monocytogenes* [49]. Conjugating bacterial targeting ligands is a potential strategy to enhance the antimicrobial effect and decrease systemic toxicity.

Hajiahmadi and colleagues developed and evaluated in vivo the therapeutic effect of a targeted vancomycin-encapsulated liposome after topical administration. Lysostaphin was coupled at liposome surface for specifically microbial targeting. Lysostaphin is an enzyme that binds to the peptidoglycan of *S. aureus* cell wall, leading to its disruption. This targeted approach enabled the immobilization of the pathogen, disrupting its cell wall and releasing the antibiotic within bacteria. Lysostaphin conjugated at liposomes surface displayed higher binding rate and bacterial effect than non-conjugated liposomes [70].

The treatment of bone infections is also a huge challenge, requiring prolonged use of antibiotics and characterized by a diminished rate of success. A major issue is the reduced vascular supply of necrotic and infected bone, hindering the antibiotic capacity to reach the infected bone at inhibitory concentrations [71]. As such, an antibiotic local administration associated to nanoplatforms is an attractive approach. Indeed, some drug delivery systems for the treatment of this pathology associated to medical devices implantation, have already been approved by FDA, such as bone cements and PMMA beads containing gentamicin [72,73,74]. However, these systems have been approved only for use in a second stage of a surgical revision. In addition, they still need to be optimized, since one of the major disadvantages is the low release rate of loaded antibiotics [22,75]. The use of liposomal formulations for this type of infections constitutes an appealing alternative strategy. Several studies have been conducted with antibiotic-loaded liposomes to reach infected bones caused by MDR strains. Different antibiotics, such as gentamicin, vancomycin, ceftazidime and dicloxacillin, among others, were incorporated in liposomes using different lipid compositions and the so developed formulations were tested in MRSA bone infections (reviewed in [22]). Again, the development of liposomes capable of targeting, in this case the bone, can also be achieved. Coating drug delivery systems with calcium phosphate or their derivatives is often performed due to calcium phosphate biocompatibility and ability to bind to bone tissue [76,77]. Hui and co-workers developed a calcium sulfate scaffold incorporating gentamicin-loaded in positively charged liposomes with sustainable release profiles. This system promoted the complete sterilization of bone tissues in a surgical implantation rabbit model infected with a *S. aureus* strain, a therapeutic effect that was not achieved when free or liposomal gentamicin were intravenously administered [78].

Bacterial meningitis is another pathology that requires noteworthy attention in terms of antibiotic delivery. Meningitis is an infection of the central nervous system (CNS) characterized by inflammation of the meninges, the protective membranes of the brain and spinal cord [79]. It is considered one of the most severe forms of infectious diseases worldwide due to the high levels of mortality and neurological sequelae among surviving patients. Although it can be caused by different microorganisms, the two most common bacterial agents are *S. pneumoniae* and *Neisseria meningitidis*, with the first being responsible for over two-thirds of the cases in Europe and USA (61%) [80,81,82]. A major issue in the clinical management of meningitis treatment is the poor antibiotic penetration through the blood–brain barrier (BBB) [81,83,84]. Essentially, 98–100% of large-protein drugs and >98% of small-molecule drugs do not cross the BBB [83]. For this reason, no matter how efficient the therapy is in clearing the pathogen, if it is unable to penetrate into the cerebrospinal fluid (CSF), its effect will be limited. One of the strategies used is to increase the systemic dose of the antibiotic. However, for the majority of the antibiotics, the subsequent increase of the toxicity levels turns this approach unsuitable and in certain cases not feasible (e.g., fluoroquinolones) [80,84]. The only alternative is to perform an intrathecal administration of the antibiotic, an invasive technique with low clinical support due to variability of patient outcomes [84]. In addition, the clinical situation can be more complicated if the infection is caused by a multidrug-resistant pneumococcus, leading to a delay in the implementation of the appropriate antibiotic and consequent poor therapeutic outcome [81,85].

Several drug-delivery approaches to cross the BBB have been investigated with liposomes unique characteristics, being considered a nanoplatform system of choice for the treatment of CNS infections. A major feature is the easy surface modification of liposomes enabling the improvement of drug translocation across the BBB. This can be achieved by either non-specific or specific targeting. Non-specific targeting is attained by using positively charged liposomes. Here, electrostatic interactions between liposomes and the polyanions present at the BBB leading to an adsorptive-mediated endocytosis [86]. Joshi and co-workers evaluated the delivery of negative, neutral and positively charged liposomes to the brain by intraarterial injection. Positively charged liposomes demonstrated the highest uptake into brain parenchyma including glioma tissue. This work revealed the capacity of positively charged liposomes to deliver molecules into the brain tissue after intraarterial injection through an intracarotid route [87,88]. However, this approach generally required the administration of high doses to reach the brain [86]. On the other hand, surface functionalization methodologies have enabled specific targeting across the BBB, improving the pharmacokinetic and biodistribution of drug-loaded liposomes into the brain. The use of ligand-targeted liposomes towards brain endothelial cells receptors has been explored, resulting in BBB translocation by receptor-mediated transcytosis [86,88,89]. Examples of these ligands include cationized albumin, OX26 monoclonal antibody to the transferrin receptor and monoclonal antibodies to the insulin receptor [88]. Loureiro et al. developed pegylated immunoliposomes with OX26Mab for targeting the transferrin receptor. Functionalized liposomes demonstrated the ability to be internalized in in vitro porcine brain capillary endothelial cells and were able to reach the brain in animal models [90]. Despite the lack of research regarding the validation of liposomes for brain infections, these studies demonstrated the potent ability of targeted liposomes to cross the BBB and deliver the incorporated antimicrobial agents in the brain, resulting in improved therapeutic effect [91].

### 4.3. Improved Bactericidal Potency and Efficacy

One of the paramount features of antibiotic loaded liposomes is the enhanced antibacterial activity when compared to the respective antibiotic in the free form. Indeed, several studies have described liposomal formulations of antibiotics with improved potency even towards resistant strains. For example, a reduction of the MIC of liposomal ciprofloxacin and gentamicin, in comparison to the free drug, against most common resistant bacteria, such as *P. aeruginosa*, *K. pneumoniae* and *E. coli*, has been observed [92,93,94]. In these studies, the authors hypothesized that the improved antimicrobial activity of these formulations was due to the efficient and extensive interaction of the liposomes with the outer membrane of the bacterial cell. Due to the similarity of the liposome structure and composition to the bacterial membrane, they have the unique capacity to interact with the bacteria, stimulate fusion with the cell membrane, enable a high antibiotic delivery into the bacteria and potentially overcoming antibiotic resistance mechanisms [13].

To accomplish this interaction, the design and optimization of liposomal formulations are crucial stages of the development process. It has been previously reported that cationic liposomal formulations generally exhibit higher antibacterial activity than anionic or neutral formulations, regardless of the incorporated antibiotic [93]. As mentioned above, this is easily explained by the fact that cationic liposomes tend to bind electrostatically at the Gram-negative bacteria outer membrane [13,18,93]. Besides the lipid charge, the fluidity or fusogenic properties of liposomes have a role in improving liposome–bacteria interactions [17,95,96,97]. In this way, the liposome–bacteria fusion process depends on the lipid composition presence of fusogenic agents at liposomal surface (i.e., charged organic compounds and metal ions) and properties of the bacteria [95]. For instance, several studies have reported an effective interaction between liposomes containing DPPC/DMPG (dipalmitoyl phosphatidyl choline/dimyristoyl phosphatidyl glycerol), a popular lipid composition commonly designated “fluidosomes”, and the bacterial membrane [17,24,95,98]. Indeed, Sachetelli et al. observed that this type of liposomes fused with the outer membrane of *P. aeruginosa*, releasing the entrapped antibiotic (tobramycin) directly to the periplasmic space and inducing a bactericidal effect at sub-MIC concentrations. Additionally, it has been demonstrated that the bactericidal effect of a liposomal formulation was improved when the fusogenic lipid DOPE (dioleoyl phosphatidyl ethanolamine) was included in the lipid composition [97,99]. Nicolosi et al. showed that the encapsulation of vancomycin in fusogenic liposomes inhibited the growth of Gram-negative bacterial strains, an effect that was not observed when free antibiotic or non-fusogenic liposomes were used against the same strains [17]. Both approaches promoted a higher degree of fusion between liposomes and the bacterial cells, resulting in increased amount of the antibiotic within bacteria [17,95,96]. Furthermore, in vitro experiments carried out by Drulis-Kawa et al., in which a single cationic and fluid liposomal formulation was tested against several *P. aeruginosa* strains, demonstrated that specific structures of the bacteria surface also tend to strengthen liposome–bacteria interactions [62]. Hence, in addition to the liposomal composition (surface charge and fluidity), the bacterial surface patterns (e.g., global surface charge, outer membrane proteins, hydrophobic properties, LPS structure) also influence the affinity between liposomes and bacteria. This opens up a number of new possibilities for the development of specific antimicrobial strategies against bacterial pathogens.

### 4.4. Overcoming Bacterial Resistance Mechanisms

**Figure 3:**
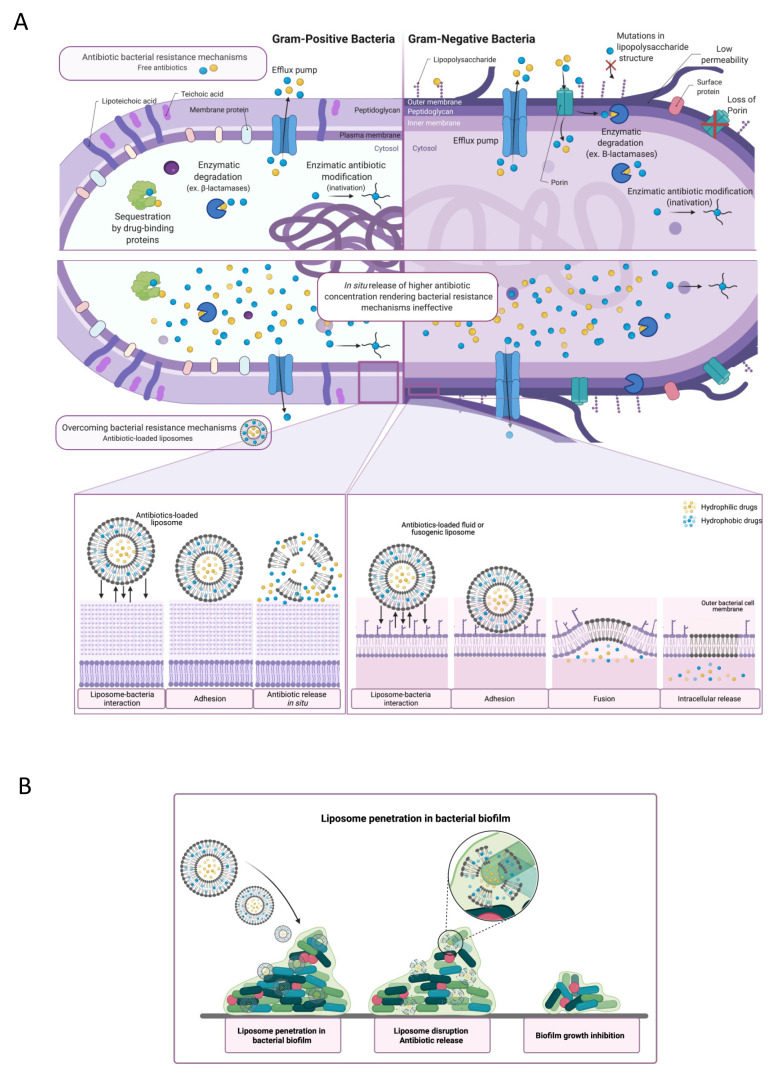
Schematic representation of antimicrobial resistance mechanisms that could be overcome by the use of antibiotic incorporated liposomes. (**A**) There are several mechanisms of antibiotic resistance, including loss of porins, which reduce the antibiotic entrance; sequestration of the antibiotics by drug binding proteins, blocking its interaction with the target; enzymatic degradation and enzymatic antibiotic modification, which alter the antibiotic making it incapable of inducing its effect; and efflux pumps which expels the antibiotic out of the cell. Blue and yellow spheres indicate antibiotics. The encapsulation of antibiotics provides the delivery of a higher antibiotic concentration within the bacteria infection site possibly stimulating the fusion with the bacterial membrane depending on the lipid composition. By increasing the antibiotic concentration, the resistance mechanisms depicted become obsolete, unable to fully block the antibiotic action. Mechanisms not shown include target modification, target bypass and antibiotic target protection. (**B**) Liposome penetration in biofilm: biofilms are considered a resistance mechanism due to the lower penetrability of the antibiotic in the extracellular matrix. Antibiotic-loaded liposomes have the ability to interact with bacteria organized in biofilm, enabling the antibiotic delivery within its structure.

Evidence is increasingly suggesting that the incorporation of antibiotics within liposomes may help to overcome certain bacterial resistance mechanisms by modulating the liposome–bacteria interactions [9,18]. For example, the outer membrane of Gram-negative bacteria is a complex barrier that can limit the internalization or change the interaction of antibiotics with the bacterial wall, being a major source of emergence resistances [13]. Nevertheless, as mentioned above, liposome may stimulate fusion with the bacterial membrane (Figure 3), promoting its structural disruption and potentially reversing its low permeability [13,17,18,64]. This fusion process can be further optimized by enhancing the fluidity of liposomes or by including fusogenic phospholipids in their composition, as discussed above. Some examples of liposomal formulations developed for MDR pathogens are depicted in Table 2.

The liposome–bacteria fusion could be a promising approach to overcome non-enzymatic drug resistance [93]. This has been studied in particular for *P. aeruginosa* strains, as their resistance mechanisms are mainly associated with low and non-specific permeability of its outer membrane and/or the presence of efflux pump systems [13,93,95]. For instance, Mugabe et al. reported that aminoglycoside-loaded liposomes could successfully treat infections caused by resistant clinical strains of *P. aeruginosa*. In their studies, the bacteria exposed to antibiotic liposomal formulations revealed higher antimicrobial susceptibility than those exposed to the free drug [92]. Another group observed that resistant *P. aeruginosa* strains treated with a fluid liposome-entrapping polymyxin B presented lower MICs and higher levels of antibiotics within the bacterial cells when compared to the free antibiotic [100]. Thus, the liposomal formulations were able to overcome one of the most efficient impermeable barriers responsible for bacterial resistance.

Additionally, antibiotics encapsulated in liposomes were able to circumvent bacterial resistance related to enzymatic hydrolysis [97]. Although this strategy has been less explored, Nacucchio et al. demonstrated that the encapsulation of piperacillin in liposomes prepared with phosphatidyl choline and cholesterol was able to protect the antibiotic against the hydrolysis by staphylococcal β-lactamases, thus retaining its antibacterial activity [101]. The design of liposome-encapsulated antibiotics with specific properties to circumvent enzymatic degradation is an interesting feature to be explored particularly against enteric rods, as their mechanisms of resistance are more often enzymatic [93].

The improved effect of liposomes has also been demonstrated for MDR intracellular pathogens such as *Mycobacterium tuberculosis*. It is known that *M. tuberculosis* can induce a long-term infection in humans mainly due to their ability to infect and persists in macrophages further complicating the eradication of this bacteria. In this particular type of infection liposomes constitute a promising therapy since they have an inherent tendency to be taken up by macrophages. In fact, Gaspar et al. demonstrated that liposomes encapsulating rifabutin not only increased the antibiotic efficacy but also decreased the damaging inflammatory response in infected organs [102].

Finally, liposomes may constitute a disruptive approach for one of the most difficult to treat hospital acquired MDR infections, namely bacterial biofilms associated to medical devices. Biofilms by themselves act as a resistance mechanism due to the lower penetrability of the antibiotic in the extracellular matrix. If the biofilm involves a multidrug-resistant strain such as methicillin resistant *Staphylococcus aureus* (MRSA), the infection may become chronic and even untreatable. Nevertheless, in vitro and in vivo studies have demonstrated improved efficacy of liposomal formulations against biofilm associated MRSA infections [69,103,104]. In particular, a liposomal formulation co-loaded with vancomycin and ciprofloxacin allowed complete sterilization of the bone in a *S. aureus* osteomyelitis model, showing this strategy has high therapeutic potential against these life-threatening infections [105].

**Table 2:** Liposomal formulations developed for MDR pathogens.

Pathogen	Emerging Resistance Patterns	Formulations Developed		Effect	Ref.
		Active Compound	Lipid Composition		
*Acinetobacter baumannii*	Carbapenem Polymyxin	Polymyxin B	Chitosan–DPPC:DSPE:Chol Chitosan–DPPC:DSPE:Chol with USMB (DPPC:DSPE:Chol)	The combination of the two systems revealed an antibacterial synergetic effect that could almost eliminate the biofilm-producing bacterium.	[106]
		Fusidic acid	DOPE:DPPC:CHEMS DPPC:Chol	An increased antibacterial effect of fusogenic liposomes (DOPE:DPPC:CHEMS) against clinical isolates in comparison to non-fusogenic formulation (DPPC:Chol) was observed (MICs of 37.5–300.0 µg/mL versus >833.0 µg/mL). Free fusidic acid did not present antibacterial effect against Gram-negative bacteria.	[107]
		Vancomycin	DOPE:DPPC:CHEMS DPPC:Chol	Fusogenic liposomes (DOPE:DPPC:CHEMS) displayed MICs of 6–12.5 µg/mL against clinical isolates, while free vancomycin and non-fusogenic formulation (DPPC:Chol) showed no antibacterial activity.	[17]
		Polymyxin B	DPPC:Chol POPC:Chol	Higher incorporation parameters for DPPC:Chol were achieved. MIC was 16-fold lower for liposomal formulation than for free antibiotic.	[100]
*Pseudomonas aeruginosa*	Carbapenem	Amikacin Gentamicin Tobramycin	DPPC:Chol	With liposomal formulations, MICs have been maintained or reduced against all tested clinical isolates, for all antibiotics incorporated in relation to respective free antibiotics (MICs reductions were antibiotic- and strain-dependent: amikacin, 2–64-fold; gentamicin, 2–64-fold; tobramycin, 1–128-fold).	[92]
		Polymyxin B	DPPC:Chol POPC:Chol	Higher incorporation parameters for DPPC:Chol were achieved. MICs against clinical isolates were 4–32-fold lower for liposomal formulation in relation to free antibiotic. Liposomal formulation promoted the antibiotic penetration into a resistant strain in higher extent than free form.	[100]
		Gentamicin	DMPC:Chol	MICs against clinical isolates and a laboratory strain were 2–16- and 4-fold lower, respectively, for liposomal gentamicin in comparison with free form. Time–kill values of liposomal formulation were equivalent to the free antibiotic, for the laboratory strain and one clinical isolate, while for the other clinical isolate the bactericidal effect was achieved at 4× MIC for liposomal formulation and free gentamicin, after 6 and 24 h, respectively.	[108]
		Norfloxacin	PCT1–EPC:Chol:α tocopherol PCT2–EPC:Chol:α tocopherol	An increased antibacterial effect against a multi-resistant strain for both formulations in comparison with free antibiotic was achieved (MIC of 3.2 µg/mL versus >30.0 µg/mL). No toxic effects were observed for any of the formulations, evaluated through an in vivo embryo chicken model.	[109]
		Ofloxacin	DMPC:Chol:DP DMPC:Chol:DPPS DMPC:Chol:DPPE DMPC:Chol:DPPA	After a susceptibility screening against reference strains of all developed formulations, DMPC:Chol:DP and DMPC:Chol:DPPS were chosen for further studies. An increased antibacterial effect against clinical isolates resistant to quinolones, mainly with DMPC:Chol:DP formulations was observed, resulting in MICs of 2–4-fold lower than free antibiotic. Higher intracellular antibiotic concentrations were obtained for both strains tested, when antibiotic was loaded in DMPC:Chol:DP.	[110]
*Enterobacteriacea*	Carbapenem ESBL^+^ Fluoroquinolones	Cefepime	EPC:Chol EPC:Chol:12NBr DOPE:12NBr	The formulation EPC:Chol:12NBr demonstrated higher incorporation parameters and, thus, was used for antibacterial study. Cefepime-loaded liposomes presented similar antibacterial activity to its free form, against an *E. coli* strain.	[111]
		Azithromycin	EPC:EPG: EPC:HSPC-3 EPC:EPG:HSPC-3 EPC:Pg EPC:EPG:Pg EPC:SLPC:-80:Pg EPC:EPG:SLPC-80:Pg	Liposomes incorporation parameters and stability assays promoted the selection of EPC:HSPC-3, EPC:Pg and EPC:SLPC:-80:Pg formulations for further experiments. MIC_50_ for all strains tested, were similar for liposomal formulations and for free antibiotic, while against bacteria in biofilm form the activity was lipid composition-dependent. Antibiotic-loaded EPC:EPG:HSPC-3 demonstrated the lower MBIC_50_ against the *E. coli* k-12 strain (8-fold lower in relation to free antibiotic).	[112]
		Ofloxacin	DMPC:Chol:DP DMPC:Chol:DPPS DMPC:Chol:DPPE DMPC:Chol:DPPA	After a susceptibility screening against reference strains of all developed formulations, DMPC:Chol:DP and DMPC:Chol:DPPS were chosen for further studies. MICs against *E. coli* clinical isolates were 4-fold lower for both formulations in relation to free antibiotic. Higher intracellular antibiotic concentrations were achieved when antibiotic was loaded in DMPC:Chol:DP.	[110]
		Norfloxacin	PCT1–EPC:Chol:α tocopherol PCT2–EPC:Chol:α tocopherol	An increased antibacterial effect against an *E. coli* strain, mainly with PCT1–EPC:Chol:α tocopherol formulation was observed, resulting in a MIC 9-fold lower than free antibiotic. In case of *Salmonella* strains, PCT2–EPC:Chol:α tocopherol presented the highest antibacterial effect with MICs of 2–17- and 16–42-fold lower than the other formulation and free antibiotic, respectively. No toxic effects were observed for any of the formulations, evaluated though an in vivo embryo chicken model.	[109]
		Polymyxin B	DPPC:Chol POPC:Chol	Higher incorporation parameters for DPPC:Chol were achieved, thus further studies were conducted with this formulation. MICs against *E. coli* and *K. pneumoniae* were 8–16- and 16-fold, respectively, for the liposomal formulation in comparison with free polymyxin B.	[100]
		Ciprofloxacin	DPPC:Chol DSPC:Chol SM:Chol	The SM:Chol formulation presented higher circulation lifetime than the remaining formulations. In this way, the efficacy of antibiotic-loaded SM:Chol was evaluated in a *Salmonella typhimurium* infection model, resulting in viable bacteria 10^3^–10^4^-fold lower in the livers and spleens of infected mice than the free antibiotic.	[113]
*Staphylococcus aureus*	Methicillin Vancomycin	Ofloxacin	DMPC:Chol:DP DMPC:Chol:DPPS DMPC:Chol:DPPE DMPC:Chol:DPPA	After a susceptibility screening against reference strains of all developed formulations, DMPC:Chol:DP and DMPC:Chol:DPPS were chosen for further studies. An increased antibacterial effect against *S. aureus* clinical isolates, mainly for DMPC:Chol:DPPS, was observed, with values 3- and 4-fold lower than free antibiotic.	[110]
		Piperacillin	PC:Chol	Antibiotic incorporated in liposomes inhibited 3-fold higher a *S. aureus* clinical isolate growth, than its free form. Experiments using exogenous staphylococcal β-lactamase demonstrated that the liposomal formulation promoted the highest degree of protection against hydrolysis by staphylococcal β-lactamase.	[101]
		Vancomycin	DSPC:DcP:Chol DSPC:DMPG:Chol	MICs and MBCs against MRSA strains were 2–4- and 4-fold lower, respectively, for both formulations in relation to free antibiotic. The DSPC:DcP:Chol formulation showed the highest efficacy. In a systemic MRSA murine model, the liposomal formulation displayed a higher therapeutic effect, improving kidney clearance by 1-log in comparison with free antibiotic.	[69]
		Vancomycin	DSPC:Chol DSPC:Chol:DSPE-PEG	At the highest antibiotic concentration tested, DSPC:Chol formulation (non-pegylated liposomes) reduced the intracellular MRSA growth inside macrophages in approximately 2- and 3-fold higher in relation to pegylated formulation (DSPC:Chol:DSPE-PEG) and free antibiotic, respectively.	[103]
		Azithromycin	Lipoid S75 Lipoid S75:SDCh Lipoid S75:Pg DPPC:DODAB	MIC and MBIC were maintained or reduced for all formulations in relation to free antibiotic. The DPPC:DODAB formulation presented the highest antibacterial activity against both planktonic and biofilm forms of all clinical isolates tested. The MICs and MBICs were 8–32- and 16–32-fold lower than free azithromycin. Liposomal formulations demonstrated biocompatibility with keratinocytes and fibroblasts.	[114]
		Methicillin	DOPE:DPPC:CHEMS: DSPE-PEG-MAL DOPE:DPPC:CHEMS:DSPE-PEG-Tat	Antibacterial activity reductions were observed for both formulations, especially for DOPE:DPPC:CHEMS:DSPE-PEG-Tat formulation. MICs against a MRSA strain were 3.3, 5.0 and >5.0 µg/mL for DOPE:DPPC:CHEMS:DSPE-PEG-Tat, DOPE:DPPC:CHEMS:DSPE-PEG-MAL and free methicillin, respectively.	[115]
*Helicobacter* *pylori*	Clarithromycin	Ampicillin Metronidazole	DPPC:Chol:NBD-PC DPPC:Fuc-E4-Chol:Chol:NBD-PC Epikuron 170:Chol:NBD-PC Epikuron 170:Fuc-E4-Chol:Chol:NBD-PC	Liposome–bacteria interaction results obtained by epifluorescence microscopy demonstrated to be strain- and lipid composition-dependent. Formulations without Epikuron 170 displayed superior interaction levels in both strains tested. However, DPPC:Fuc-E4-Chol:Chol:NBD-PC showed the highest interaction levels in the strain that express the *babA2* gene (*H. pylori* 17875), due to the specifically link between the BabA2 protein and the fucose at the surface of liposomes.	[116]
		Amoxicillin	LC:Chol:DDAB PCT-LC:Chol:DDAB	Although both formulations presented similar antibacterial effect, the experimental assays developed in this study evidenced a specific interaction of PCT-coating liposomes with mucins and surface structures of bacteria.	[117]
*Campylobacter*	Fluoroquinolones	Norfloxacin	PCT1–EPC:Chol:α tocoferol PCT2–EPC:Chol:α tocoferol	An increased antibacterial activity against a *Campylobacter jejuni* strain, mainly with PCT–EPC:Chol:α tocoferol formulation was observed. MIC was 10-fold lower than free antibiotic. No toxic effects were observed for any of the formulations, evaluated in an in vivo embryo chicken model.	[109]
*Streptococcus pneumoniae*	Penicillin	Vancomycin	DOPE:DPPC:CHEMS: DSPE-PEG-MAL DOPE:DPPC:CHEMS:DSPE-PEG-Tat	MICs were approximately 2-fold lower for both formulations than respective free antibiotic. For the lowest concentrations tested (0.6 µg/mL) the formulation. DOPE:DPPC:CHEMS:DSPE-PEG-Tat displayed more favorable results, with a reduction of viable bacteria of approximately 1- and 2-fold in relation to the other formulation and to free vancomycin, respectively.	[115]

DPPC, dipalmitoyl phosphatidyl choline; DSPE, distearoyl phosphatidyl choline; Chol, cholesterol; DOPE, dioleoyl phosphatidyl ethanolamine; CHEMS, cholesteryl hemisuccinate; POPC, palmitoyloleoyl phosphatidyl choline; DMPC, dimyristoyl phosphatidyl choline; EPC, egg phosphatidyl choline; PCT, pectin from apple; PCT1, pectin from apple, found in the aqueous phase that surrounds the liposomes; PCT2, pectin from apple, distributed in the water phase inside and outside the liposomes; DPPS, dipalmitoyl phosphatidyl serine; DP, dihexadecyl hydrogen phosphate; DPPE, dipalmitoyl phosphatidyl ethanolamine; DPPA, dipalmitoyl phosphatidic acid; 12NBr, *N,N,N*-triethyl-*N*-(12-naphthoxydodecyl)ammonium surfactant; EPG, egg phosphatidyl glycerol; HSPC-3, hydrogenated soybean phosphatidyl choline; SLPC-80, monoacyl soybean phosphatidyl choline; PEG, propylene glycol; PC, soybean phosphatidyl choline; DSPC, distearoyl phosphatidyl choline; SM, Egg sphingomyelin; DcP, dicethyl phosphate; DMPG, dimyristoyl phosphatidyl glycerol; DSPE-PEG, distearoyl phosphatidyl ethanolamine covalently linked to poly(ethylene glycol) 2000; Lipoid S75, soybean lecithin containing 75% phosphatidyl choline; SDCh, sodium deoxycholate; DODAB, dioctadecyldimethyl ammonium bromide; DSPE-PEG-MAL, distearoyl phosphatidyl ethanolamine covalently linked to poly(ethylene glycol) 2000 linked to maleimide; Tat, cell penetrating peptide (Cys-Tyr-Gly-Arg-Lys-Lys-Arg-Arg-Gln-Arg- Arg-Arg-NH_2_); NBD-PC, fluorescent nitrobenzoxa diazolyl label linked to phosphatidylcholine; Fuc-E4-Chol, Cholesteryl tetraethylene glycol fucose; Epikuron 170, phosphatidyl choline > 72%, phosphatidyl ethanol amine > 10%, phosphatidyl inositol < 3%, lyso phosphatidyl choline < 4% and free fatty acids 10%; LC, lecithin; DDAB, di-dodecyldimethylammonium bromide; MBIC, minimum biofilm inhibitory concentration; MIC, minimum inhibitory concentration; MIC_50_, minimum inhibitory concentration that inhibited bacterial growth by 50%; USMB, ultrasound microbubbles.

## 5. Conclusions

Modern medicine is now facing a major challenge for the treatment of bacterial infections due to the emergence of pathogens with resistance to currently available antibiotics. To overcome this problem, extensive research is focused in developing new antibiotic delivery strategies to improve its antibacterial efficacy, among which liposomes are considered one of the most promising delivery nano-platforms. Their wide versatility in terms of structure and lipid composition allows the design of numerous liposomal formulations, with improved pharmacokinetics and pharmacodynamics properties [13]. On the other hand, they are able to protect the entrapped drug from premature enzymatic and immunological inactivation [118] and deliver the antibiotic directly to the infected site, tissue or pathogen in a controlled and sustained manner, limiting its distribution to healthy tissues and minimizing possible toxic side effects [13,119]. Furthermore, liposome lipid bilayers may allow a direct interaction or fusion with the bacterial cell walls, increasing antibiotic concentration within the bacteria and thus contributing to an improvement of the therapeutic effect of the loaded antibiotic [13]. Furthermore, liposome-encapsulated antibiotics have been shown to overcome certain microorganism resistance mechanisms, such as impermeable outer membrane, efflux mechanisms and enzymatic degradation. In conclusion, considering their unique physicochemical properties and advantages as antibiotic carriers, liposomes constitute a highly promising strategy to restore treatment options against currently untreatable bacterial infections.

## Figures and Tables

**Table 1:** Selected liposomal formulations and lipid-based vaccines FDA approved for human use or in clinical trials for the treatment of infectious diseases.

Commercial Name	Company	Active Compound	Lipid Composition	Indication
Ambisome^®^	Gilead Sciences/ Fujisawa Healthcare	Amphotericin B	HSPC:DSPG:Chol	Fungal infections
Amphotec^®^/Amphocil^®^	Ben Venue Laboratories	Amphotericin B	Cholesteryl sulfate	Fungal infections
Abelcet^®^	Sigma-Tau Pharmaceuticals	Amphotericin B	DMPC:DMPG	Fungal infections
Epaxal^®^	Crucell	Formalin-inactivated Hepatitis A virus	DOPC:DOPE	Hepatitis A
Inflexal^®^	Crucell	Inactivated hemaglutinine of Influenza virus	DOPC:DOPE	Influenza
Arikayce^®^	Insmed, Inc.	Amikacin	DPPC:Chol	Mycobacterium avium complex (MAC) lung disease
Arikace ^TM^	Transave, Inc.	Amikacin	DPPC:Chol	Pseudomonas aeruginosa infections (cystic fibrosis)
RTS,S/AS01	GlaxoSmithKline	Recombinant fusion of *P. falciparum* circumsporozoite protein and Hepatitis B surface antigen	MPL:DOPC:Chol	Malaria
ALIS	Insmed, Inc.	Amikacin	DPPC:Chol	Nontuberculous Mycobacterial lung infection
Vaxisome	NasVax	Inactivated Influenza virus	CCS	Influenza
JVRS-100	Juvaris BioTherapeutics	Inactivated Influenza virus	CLDC:Chol	Influenza
Nyotran	Aronex Pharmaceuticals	Nystatin	DMPC:DMPG:Chol	Fungal infections
CAF01	Statens Serum Institut	Subunit protein antigen Ag85B-ESAT, DDA, TDB	DODAB:TDB	Tuberculosis
Vaxfectin	Vical	Plasmid DNA-encoded influenza proteins	VC1052:DPyPE	Influenza
MPER-656 Liposome Vaccine	National Institute of Allergy and Infectious Diseases (NIAID)	Immunogenicity of an HIV-1 gp41 MPER-656	DOPC:DOPG	HIV infections

DPPC, dipalmitoyl phosphatidyl choline; DSPG, distearoyl phosphatidyl glycerol; Chol, cholesterol; DOPE, dioleoyl phosphatidyl ethanolamine; HSPC, hydrogenated soybean phosphatidyl choline; DMPG, dimyristoyl phosphatidyl glycerol; DMPC, dimyristoyl phosphatidyl choline; DOPC, dioleoyl phosphatidyl choline; DOPG, dioleoyl phosphatidyl glycerol; DPPC, dipalmitoyl phosphatidyl choline; CCS, ceramide carbamoyl-spermine; MPL, monophosphoryl lipid A; CLDC, oleoyl oxy ethyl oleyl hydroxyethyl imidazolinium-chloride; DODAB, dioctadecyldimethyl ammonium bromide; TDB, trehalose 6,6′-dibehenate; VC1052, aminopropyl dimethyl tetradecenyloxy propanaminium bromide; DPyPE, diphytanoyl phosphatidyl ethanolamine.
